# Engineering 3D degradable, pliable scaffolds toward adipose tissue regeneration; optimized printability, simulations and surface modification

**DOI:** 10.1177/2041731420954316

**Published:** 2020-09-16

**Authors:** Shubham Jain, Mohammed Ahmad Yassin, Tiziana Fuoco, Hailong Liu, Samih Mohamed-Ahmed, Kamal Mustafa, Anna Finne-Wistrand

**Affiliations:** 1 Department of Fibre and Polymer Technology, KTH Royal Institute of Technology, Stockholm, Sweden; 2 Tissue Engineering Group, Department of Clinical Dentistry, Faculty of Medicine, University of Bergen, Hordaland, Norway; 3 Department of Solid Mechanics, KTH Royal Institute of Technology, Stockholm, Sweden

**Keywords:** 3D Printing, poly(L-lactide-co-trimethylene carbonate), polydopamine, finite element analysis, mesenchymal stem cells, adipose tissue regeneration

## Abstract

We present a solution to regenerate adipose tissue using degradable, soft, pliable 3D-printed scaffolds made of a medical-grade copolymer coated with polydopamine. The problem today is that while printing, the medical grade copolyesters degrade and the scaffolds become very stiff and brittle, being not optimal for adipose tissue defects. Herein, we have used high molar mass poly(L-lactide-co-trimethylene carbonate) (PLATMC) to engineer scaffolds using a direct extrusion-based 3D printer, the 3D Bioplotter^®^. Our approach was first focused on how the printing influences the polymer and scaffold’s mechanical properties, then on exploring different printing designs and, in the end, on assessing surface functionalization. Finite element analysis revealed that scaffold’s mechanical properties vary according to the gradual degradation of the polymer as a consequence of the molar mass decrease during printing. Considering this, we defined optimal printing parameters to minimize material’s degradation and printed scaffolds with different designs. We subsequently functionalized one scaffold design with polydopamine coating and conducted in vitro cell studies. Results showed that polydopamine augmented stem cell proliferation and adipogenic differentiation owing to increased surface hydrophilicity. Thus, the present research show that the medical grade PLATMC based scaffolds are a potential candidate towards the development of implantable, resorbable, medical devices for adipose tissue regeneration.

## Introduction

For patients suffering from large adipose tissue defects—for example, after surgical removal of breast tissue, traumatic injury, or severe deep burns—tissue reconstruction remains one of the foremost clinical challenges for plastic and reconstructive surgeons.^1,2^ There is an increasing demand for adequate medical implants, since these large adipose tissue defects do not regenerate spontaneously and require a high volume of adipose tissue to maintain the shape and restore functionality.^3^ The commercially available fillers such as hyaluronan, fibrin, and viscoelastic hylan gels have been used for adipose tissue regeneration.^4–6^ While these methods offer some degree of clinical success, they nevertheless present some shortcomings such as shrinkage and loss of implant volume.^7,8^

During adipose tissue regeneration, the biomechanical properties of the scaffold play a role in regulating the formation of the adipose tissue matrix and subsequent regeneration. The scaffold must be able to handle external and physiological loads in order to prevent stress on the nascent tissue over the long term.^9^ Native tissue formation can be disrupted by mechanical stresses, and excess stress can inhibit the process of adipogenesis.^10,11^ Moreover, scaffolds should be able to degrade over time making room for the regenerated tissue, while maintaining their mechanical strength at approximately the same rate as tissue regeneration occurs. It has been shown that the tissue engineering constructs needs to maintain structural integrity during 9–12 months to regenerate a clinically relevant tissue volume.^9,12^

Hydrogels are widely used and show good results in soft tissue reconstruction as an alternative to current methods and fillers.^13–16^ However, the potential to scale up for large volume adipose tissue defect with adequate shape, size and structural integrity over time remains questionable. Alternatively, the use of synthetic degradable polymers with the aid of additive manufacturing/3D printing to manufacture customized 3D scaffolds is a promising approach to regenerate large portion of adipose tissue. Synthetic polymers give the possibility to fine tune the physical properties of scaffolds providing functional support for longer period. In addition, 3D printing allows fabrication of 3D scaffolds with microporous architecture, which serves as a solid template to promote adipose tissue regeneration and to stimulate cellular interactions and subsequently tissue formation.^3,9,17,18^

Owing to their easy accessibility and good mechanical properties, aliphatic polyesters especially polylactide (PLA) and polycaprolactone (PCL) have widely been explored in several tissue engineering applications.^19–22^ Aliphatic polycarbonates are being explored as another class of material that is degradable and resorbable.^23^ Poly(trimethylene carbonate) (PTMC) and its copolymers have great potential especially in soft tissue regeneration due to their flexibility, non-toxic and non-acidic degradation products.^24^ Apart from providing pliability, another important advantage of using PTMC is that it degrades by a mechanism of surface erosion. Surface erosion enables a longer retention of the material integrity, with a gradual and less abrupt decrease of the mechanical properties in comparison to bulk hydrolysis process, since the molar mass and molecular structure of the bulk of the material is not affected.^24,25^ In contrast, polyesters undergo bulk degradation and lose their mechanical properties faster compared to surface eroding materials. Given a specific degradation process, the degradation rate is influenced by many parameters, for example, molar mass and scaffold design.^26^ We have recently shown that during hydrolytic degradation, PLLA fibers lost mechanical integrity in 15 weeks, whereas fibers prepared with its copolymer containing 20 mol% of trimethylene carbonate (TMC) underwent a more gradual degradation and maintained their mechanical properties for up to 30 weeks, despite having an initial lower degree of crystallinity.^27^

To date, only few researchers have leveraged 3D printed microporous scaffolds for adipose tissue engineering from synthetic degradable polymers^9,28^ because after printing scaffolds usually become stiff and brittle, which are not suitable for in vivo applications. Interestingly, Chhaya et al. have used poly(D,L-lactide) based customized scaffolds to replicate breast shape; obtained from a laser scan, once implanted in a mouse model the scaffolds showed suitability to engineer adipose tissue.^9^ To improve the mechanical behavior, elasticity and degradation of the printed scaffolds, they subsequently prepared a scaffold using poly(D,L-lactide-co-caprolactone).^29^ Still, there is a need to develop another class of materials which can primarily be used for soft tissue engineering. Despite being flexible and soft, researchers have not utilized PTMC and its copolymer in combination with 3D printing to develop scaffolds for adipose tissue regeneration. It is well known that by varying the combination, ratio, and primary structure of the monomers we can fine-tune the mechanical properties and degradation profile of the copolymer. Copolymerization of lactide with TMC has been shown to generate improved mechanical properties and pliability in comparison to PLLA toward soft tissue engineering applications.^24,30^

Therefore, herein, we used poly(L-lactide-co-trimethylene carbonate) (PLATMC) and engineer a soft, pliable, degradable, medical-grade 3D printed scaffold with improved cell-material interactions to promote adipose tissue regeneration. We know from our previous research^31^ that when printing degradable polymers at high temperature, the polymers’ properties—such as crystallinity and molar mass—undergo changes that can subsequently influence the mechanical properties of the scaffolds. Therefore, herein, the polymer was characterized to understand their physical and chemical properties before and after the printing process. Additionally, we performed computational simulations to explore the effect of reduction in molar mass on the mechanical properties of the scaffolds.

Surface hydrophilicity is known to also play an important role in cell attachment and proliferation.^32^ In the present research, we therefore modified the surface of the printed scaffolds using polydopamine (PDA). This simple and versatile surface modification approach is based on mussel-inspired chemistry for biomaterials.^33^ Dopamine (DA) contains catechol and amine groups and polymerizes to PDA at alkaline pH. PDA can be coated easily onto the substrate in a thin layer, which can then be used as a platform for post-modification thanks to the presence of the catechol and amine groups on the surface. These can covalently bind various bioactive molecules, anticancer drugs, thiolated peptides, growth factors, or antibodies.^34–36^ Another advantage of PDA is its antibacterial properties.^37^

**Figure 1.**
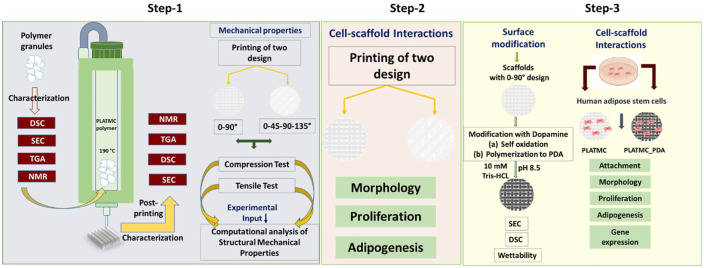
Overview of the approaches used. Step 1: establishing printing parameters and critical analysis of polymer properties before and after printing, followed by computational analysis to understand the relationship between molar mass and the structural/mechanical properties using two different designs. Step 2: Cell-scaffold interaction in two different designs. Step 3: ASC response to PLATMC and PLATMC_PDA modified 3D printed scaffolds. (Differential scanning calorimetry (DSC), Size-exclusion chromatography (SEC), Thermogravimetric analysis (TGA), and Nuclear magnetic resonance (NMR)).

With this research approach, we achieved first an understanding of how the printing process influences the PLATMC and scaffold properties, as well as the influence of scaffold design on seeding efficacy and stem cell behavior. We thereafter assessed the potential of PLATMC and PLATMC_PDA scaffolds for adipose tissue regeneration using human adipose-tissue–derived stem cells (ASC), evaluating cell attachment, proliferation, and differentiation of ASC cultivated in vitro on the unmodified and modified scaffolds. Figure 1 gives an overview of the strategies used.

## Experiment

### Materials

Medical-grade poly(L-lactide-co-trimethylene carbonate) (PLATMC) RESOMER^®^ LT 706 S was purchased from Evonik GmBh, Germany. Dopamine hydrochloride (H8502), indomethacin (I7378), dexamethasone (D4902), and insulin (I2643) were purchased from Sigma-Aldrich. The polymer was stored at 4°C according to the manufacturer’s instructions.

### 3D printing procedure and scaffold fabrication

Using the Magics software (EnvisionTEC, Germany), a 3D CAD model was designed and then sliced into different layers at a slice thickness of 80% of the inner needle diameter (ID). A slice thickness of 0.32 mm (3D Bioplotter^®^ RP, EnvisionTEC) was used for a 0.4 mm (ID) stainless steel needle, according to the manufacturer’s guidelines (EnvisionTEC). The cartridge was preheated to a designated preheating temperature of 220°C before adding 2.5 g of polymer granules to test the printability of the polymer. The polymer was then kept at this temperature for 4 min before reducing the temperature to the designated printing temperature, which was kept constant during the entire printing time. Different samples were extruded at defined time points and evaluated to assess printability.

For the in vitro biological characterization, a 4-layer rectangular sheet measuring 35×35×1 mm was printed, and 8 mm diameter scaffolds were punched out for the cell studies. Two different scaffold designs were prepared by varying the angle of rotation of the deposited strands. This can change the scaffold’s bulk mechanical property, pore geometry/size and stress distribution within the scaffolds which further could affect stem cells behavior. (1) B90, where the angle of rotation for a continuous layer was 0 and 90°. A 0.15 mm shift was set for layer 3, which means layer 3 would be shifted from the position of layer 1, and similarity layer 4 would be shifted from layer 2. This was designed using the manufacturer’s software. (2) B45: 4 different angle rotation 0-45-90 and 135° was set to fabricate the scaffolds. The gap between the strands was 0.7 mm for both designs.

### Characterization of the polymer and scaffolds

#### DSC

DSC was used to analyze the thermal properties of the polymer, such as glass transition temperature (T_g_), enthalpy of fusion (ΔH_m_) crystallization (ΔH_c_), and melting point (T_m_), both before and after printing. The DSC instrument (Mettler Toledo) was calibrated with indium and used to record thermograms. Aluminum pans were used to hold the samples. Measurements were taken in an N_2_ atmosphere at a temperature range from –20°C to +220°C with a 10°C min^−1^ temperature ramp. The data were obtained from the first heating run. The midpoint ASTM temperature was considered for *T_g_* (first cooling), and *T_m_* (first heating) as the maximum of the endothermic peak. The first heating run was used to calculate the percentage degree of crystallinity (*X_c_*) as *X_c_* (%) = [(ΔH_m_ – ΔH_c_)/ΔH_m_°] × 100, where ΔH_m_° is the enthalpy of fusion of a % crystalline PLLA and it is equal to 93.0 J g^-1^.^38^

#### SEC

Molar mass (*M_w_* and *M_n_*) and dispersity (Ð) were determined using SEC. The analysis was performed on a GPCMAX system supplied with three columns, one guard column (TGuard) and two linear mix beads (LT4000L), along with one RI detector. The eluent phase used was CHCl_3_ with a 0.5 mL min^-1^ flow rate. Flow rate fluctuations were corrected using an internal standard (toluene), and the standard curve was plotted using a narrow polystyrene standard.

#### TGA

TGA was carried out using a Mettler Toledo instrument in an oxygen atmosphere with the temperature ranging from 25 to 450°C, with a heating rate of 10°C min^−1^ and an 80 mL min^−1^ flow rate. Isothermal TGA was performed to assess the loss of mass during printing. The conditions simulated the printing conditions: PLATMC was heated under oxygen flow (80 mL min^−1^) at 220°C for 4 min, and the temperature was then reduced to 190°C and kept constant for 240 min. Finally, the temperature was raised to 450°C at a rate of 10°C min^−1^.

#### X-ray micro-computed tomography (Micro-CT)

The 3D printed scaffolds were scanned using a high-resolution cone-beam micro-CT Skyscan 1172 scanner (SkyScan 1172, Belgium). The x-ray source was set at 40kV of X-ray accelerating voltage and 200A of current. No filter was used. The “medium” camera pixel resolution (2000*2000) was used. Data sets were reconstructed using standardized cone-beam reconstruction software (NRecon, SkyScan).

#### NMR

Deuterated chloroform (CDCl3) was used to dissolve the scaffolds and proton (^1^H) and carbon (^13^C) NMR spectra were recorded using Bruker top spin software on Bruker 400 Ultrashield spectrophotometer. MestResNova software was used to analyze the spectra.

### Mechanical test and computational analysis

#### Compression tests

The compression tests were performed using an Instron 5566 universal tester on both scaffold designs. Cylindrical specimens measuring 5.5 mm height and 6.5 mm diameter (17 layers) were utilized for the tests. The specimens were compressed at a rate of 10% of their initial height per min, and then the stress-strain curve was used to calculate Young’s modulus.

#### Tensile tests

The tensile tests were performed using an Instron 5944 universal testing machine; the samples were held using pneumatic grips. We used scaffolds consisting of 4 layers (B90 and B45) measuring approximately 50 mm height, 7 mm diameter, and 1.1 mm thick with a load of 500 N at a crosshead speed of 500% of the initial specimen length per min.

#### Computational simulations and parameters

Computational simulations were carried out to investigate the structural and mechanical properties of the B90 and B45 scaffolds. A 3D description of the scaffold geometry was generated based on the structural parameters, using the ABAQUS 6.14 assembly module (Dassault Systems, France), which was then used for finite element analysis (FEA) simulations.

**Figure 2.**
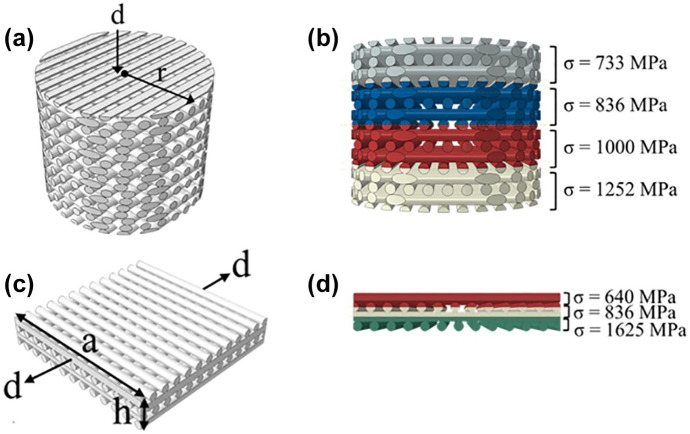
Schematic diagrams of the models in the FEA simulations: (a) compression model; (b) material assignment in the compression model; (c) tension model; (d) material assignment in the tension model, where *d* is the displacement applied on the rigid plate, *r* is the radius of the cylindrical scaffold, and *a* and *h* are the width and the height of the quadratic scaffold, respectively.

Small-strain FEA was used to simulate the structural properties of the scaffolds. For the compression model, the cylindrical scaffold sample was compressed between two rigid plates by axial compressive strain of −1% in the thickness direction, as shown in Figure 2a. For the tension model, the quadratic scaffold sample was stretched between two rigid plates by a strain of 1% along its axial direction (see Figure 2b). PLATMC was defined as a linear elastic material for the scaffold structure. Two scenarios were considered for the material assignment of the scaffold structure. The first scenario was that the material’s properties were identical throughout the whole scaffold (elastic modulus of 1625 MPa and Poisson’s ratio of 0.3):^39,40^ that is, that the loss of average number molar mass (*M*_n_) has no influence on the mechanical properties of the 3D printed PLATMC strands. The second scenario was that the material properties were variable across the scaffolds. In this scenario, the elastic modulus of the PLATMC was assumed to decrease with the loss of molecular weight in a linear correlation. Correspondingly, the material assignment for scaffolds is shown in Figure 2c and d for the tension and compression simulations, respectively. In the next section, we refer to these two scenarios as “identical” and “variable,” respectively.

A surface-to-surface contact model, with the coefficient of friction of 0.2, was used to prescribe the corresponding surface displacements. Stress and strain within the scaffold structure were predicted by solving the static Cauchy equation of motion via the commercial finite element code ABAQUS/Standard. The simulated reaction force $F_{R}$ was then used to calculate the effective compressive and tensile moduli using the following equation:

(1) $$E=F_{R}/\left(A\varepsilon \right)$$

where ε= 0.01 denotes the average strain, and the cross-sectional area A for the compression simulation = *πr*^2^ = 78.54 mm^2^ and the cross-sectional area A for the tension simulation = *ah* = 12.8 mm^2^.

### Surface modification of the 3D printed scaffolds (PDA coating)

After the 3D printed scaffolds were fabricated, they were coated with PDA. To do so, the scaffolds were immersed in 2 mg ml^-1^ of dopamine hydrochloride solution (Sigma Aldrich) which is a widely used concentration for an effective coating,^36,41^ prepared in a 10 mM Tris buffer, pH 8.5, then placed on a shaker at 100 rpm for 12 h at room temperature (RT). Dopamine undergoes self-oxidative polymerization in the presence of an alkaline pH and atmospheric oxygen, which is evidenced visually by its change in color from white to black. The coated scaffolds were then rinsed with deionized water repeatedly to remove unattached dopamine.

### Contact angle measurement

Wettability of the PLATMC and PLATMC_PDA was tested by measuring the water contact angle, placing a 3 µl MiliQ water drop randomly on the surface of films prepared from printed polymer dissolved in chloroform and measuring the water contact angle using CAM 2008 software. The measurements were taken at room temperature.

### Cell studies

#### Collection, isolation, characterization, and expansion of adipose tissue

Human adipose tissue samples from multiple donors aged between 8–14 years, were used to isolate ASC by employing the approved procedure by the regional committee for Medical and Health Research Ethics (REK) in Norway (2013/1248/REK), from patients at Haukeland University Hospital in Bergen, Norway.

As described previously,^42^ a subcutaneous adipose tissue block was used to isolate ASC. The adipose tissue block was washed three times with a 5% antibiotic solution prepared in phosphate-buffered saline (PBS) (Invitrogen USA). Afterward, it was chopped into small pieces and incubated with a digestive solution in PBS (2% antibiotic and 0.1% collagenases type I; Worthington Biochemical Corporation, USA), at 37°C for 60 min. Then a culture medium, consisting of Dulbecco’s Modified Eagle *Medium* (*DMEM) (Invitrogen*) supplemented with 10% fetal bovine serum (Hyclone GE Healthcare Life Sciences, USA) and 1% antibiotics, were added to neutralize collagenase activity. The digested fat was then centrifuged at 2000 rpm for 5 min, shaken vigorously, and recentrifuged. After that, the supernatant was discarded and the pellet dispersed in the culture medium, plated in a tissue culture flask, and held in a standard incubator (Thermo Scientific, USA) at 37°C with 5% CO_2_. Every three days, the culture medium was changed until it reaches the confluence of 75–80%. Cell morphology was monitored during expansion using an inverted microscope (Nikon Eclipse TS 100, Japan). At the confluent stage, the cells were dissociated using Trypsin-0.25% EDTA (Lonza, Switzerland), then expanded for 3–4 passages for use in the experiment. Before the cells were used for the experiment, they were characterized as mesenchymal stem cells based on expression of the surface markers; CD90, CD45, CD73, CD34, CD105, and HLR-DR (BD Bioscience, USA), as previously reported,^42^ using a cell analyzer (BD LSRFortessa, BD Bioscience).

#### Cell attachment and proliferation

The PLATMC and PLATMC_PDA scaffolds were sterilized with 70% ethanol for 30 min and repeatedly washed with PBS, then kept under an ultraviolet lamp for one hour. The scaffolds were centrifuged to remove entrapped air, then pre-soaked in culture medium overnight prior to seeding. A cell suspension of 25 µl containing 2 × 10^5^ cells was added onto each scaffold and additional medium was supplied to submerge the scaffolds, then allowed to attach for one hour. Subsequently, sufficient medium was supplied and refreshed every three days.

DNA quantification was assessed using the Picogreen assay (Thermo Scientific, USA), as described in an earlier publication.^43^ The culture medium was aspirated, and the scaffolds were washed with PBS, 0.2 ml of lysis solution (0.2 mg mL^-1^ proteinase K and 0.02% sodium dodecyl sulfate) were added and the suspension was held at 37°C for 12 h. The lysate was collected in a 96-well-plate and Picogreen dye was added to the well in equal amount of the lysate by following manufacture’s protocol. The intensity of fluorescence was measured using a microplate reader (Thermo Scientific, USA), with 485 nm as excitation wavelength and 520 nm as the emission wavelength. The cellular dsDNA content was calculated against a standard curve obtained by serial dilution of a known concentration of DNA.

Confocal microscopy was used to visually monitor cell morphology and proliferation. Scaffolds were fixed with 4% paraformaldehyde (PFA) and then washed with PBS to remove the PFA. Cells were permeabilized with 0.2% Triton-X for 10 min. The nuclei were stained using 4′,6-diamidino-2-phenylindole (DAPI) dye (14.3 mM; 15 min, Thermo scientific, USA), and the cytoskeleton was stained using Alexa Fluor 488 dye (6.6 µM; 20 min; Thermo Scientific, USA). The fixed scaffolds were sputter-coated using gold to prepare them for scanning electron microscopy (SEM) (Phenom XL desktop SEM, Thermo Scientific), then imaged at 10 kV.

#### Adipogenic differentiation

Potential adipogenic differentiation of the ASCs was assessed by culturing the cells in an adipogenic medium prepared by adding adipogenic supplements (dexamethasone (1 µM), 3-isobutyl-1-methylxanthine (IBMX) (500 µM), indomethacin (100 µM), and insulin (10 µg/ml)) to the growth medium. All these supplements were purchased from Sigma Aldrich. Adipogenic supplements were added after 72 h of cell seeding, which was taken to be day 1 of the count.

Fluorescence staining and measurement of intracellular lipid vesicles was done using the AdipoRed^TM^ assay (Lonza) by following the manufacturer’s protocol. The scaffolds were washed with PBS, placed in 48-well-plate, and incubated with 400 µl of PBS containing 12 µl of dye per well at RT for 10 min. Then images were taken using a confocal laser microscope.

#### Gene expression analysis

Adipogenesis was confirmed by assessing the expression of selected adipogenic genes (Table S1). Cells were cultivated in adipogenic medium, and RNA was isolated at days 10 and 21. The total RNA content was obtained using an RNA extraction kit (Maxwell®, Promega, USA) following the manufacturer’s protocol. The Nanodrop spectrophotometer (Nanodrop Technologies, USA) was then used to determine the quantity and purity of the extracted RNA. 300 ng of the total RNA was used to synthesize cDNA using a high-capacity cDNA reverse transcriptase kit (Applied Biosystem, USA), following the supplier’s recommended protocol. A TaqMan Fast Universal PCR master mix (Applied Biosystem) was used to perform real-time quantitative polymerase reaction (qPCR), with amplification on the StepOne^TM^ real-time PCR system (Applied Biosystem) under standard cyclic and enzymatic conditions using a 96-well thermal cycle plate. Expression of adipogenic genes, peroxisome proliferator-activated receptor gamma (PPARG), CCAAT enhancer binding protein alpha (CEBPA), lipoprotein lipase (LPL), and ADIPOQ (adiponectin, C1Q and collagen domain containing) were assessed for cell differentiation. Glyceraldehyde-3-phosphate dehydrogenase (GAPDH) was used as an internal control, and the fold changes were calculated by following the 2-^∆∆CT^ method. Table S1 lists the primer sequences used.

### Statistical analysis

Significant differences were carried out using 1-way ANOVA- analysis with Tukey’s test for multiple comparisons. Statistically, differences were considered for *p* < 0.05, and *p* < 0.01, represented by the symbols, * and **, respectively.

## Results and discussion

### Characterization of the polymer granules

We used PLATMC to fabricate soft, pliable, degradable scaffolds. The polymer granules were characterized as-received using NMR, DSC, SEC and TGA in order to have an overview of the properties (Table S2). The copolymer had a composition of 60 mol% in L-lactide and 40 mol% in TMC, as determined by ^1^H NMR and the length of the lactidyl (L_LL_) and trimethylene carbonate (L_T_) blocks, determined by ^13^C NMR were respectively 2.3 and 1.5. The copolymer was semi-crystalline, with low degree of crystallinity (X_c_ = 12%), and exhibited a melting temperature (T_m_) of 158°C and a glass transition temperature (T_g_) of 29°C.

### 3D printing procedure, parameters, and polymer behavior

We used the extrusion-based 3D printer (Bioplotter^®^) to fabricate the PLATMC scaffolds. Our previous experience^31^ using medical-grade poly(L-lactide) and its copolymers—poly(L-lactide-co-glycolide) (PLGA), poly(L-lactide-co-ε-caprolactone) (PCLA), and poly(D, L-lactide-co-glycolide) (PDLGA)—helped us to set a starting point for printing. A printing temperature of 30–40°C above T_m_ is suitable for printing, and a lower pressure range (2–6 bar) is beneficial in order to minimize the material degradation while maintaining an adequate printing speed. In addition, the preheating of the printing cartridge to a temperature higher than the printing temperature a few minutes after adding the polymer sped up the melting process and decreased degradation.

After trials and optimization, we established the printing parameters for PLATMC. We first preheated the cartridge to 220°C and added 2.5 g of polymer and held it for 4 min. After 4 min, we reduced the temperature to 190°C (printing temperature) maintaining it constant during the whole printing process. The scaffolds were fabricated using a needle with an inner diameter (ID) of 0.4 mm and outer diameter (OD) of 0.7 mm. The speed ranged from 6 to18 mm sec^-1^ and the pressure ranged from 2 to 6 bar. Pressure and speed were adjusted according to the melt flow; thus, that high resolution could be achieved.

**Figure 3.**
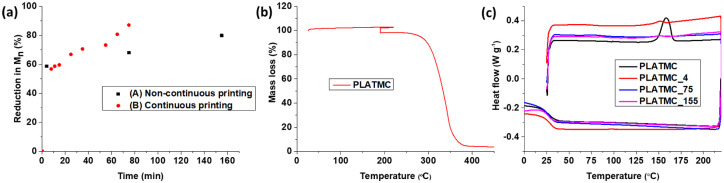
Characterization of PLATMC while printing. (a) The number average molar mass (M_n_) was determined using SEC during the printing time, at 190°C. Different samples were collected: non-continuous printing, with purging when samples were collected; and continuous printing, with samples collected until the polymer degraded or until there was no polymer left in the cartridge. (b) Mass loss determined by TGA analysis of the copolymer in an oxygen atmosphere similar to printing conditions. (c) DSC thermogram (first run) of PLATMC copolymer showing the changes in thermal properties due to printing over a period of time. The suffix X in PLATMC_X indicates the time when the sample was collected.

As shown previously,^31^ high pressure (8.5 bar) led to faster polymer degradation provoking a fast decrease of the viscosity in comparison to the low-pressure range of 2–6 bar. However, the degradation was also influenced by the polymer composition. Herein, we wanted to find a balance between pressure, speed and degradation, and evaluated therefore different printing conditions, aiming for high printability while minimizing degradation. We collected samples under two different conditions: (A) non-continuous printing, purged only when the samples were collected, and with variable pressure changed (2–6 bar); and (B) continuous printing under constant pressure, collecting samples in the meantime printing process. The temperature was constant in both conditions, and the speed was varied in order to optimize printing quality. Figure 3 and Table 1 summarize the effect of the printing parameters on physical properties of the polymer.

**Table 1.** Changes in thermal properties and molecular weight during printing, analyzed by using DSC and SEC.

Polymer	M_n_ (kg mol^-1^)	Ð	T_g_ (°C)	T_m_ (°C)	T_c_ (°C)	X_c_ (%)
PLATMC granules	109	1.6	29	158	–	12
PLATMC_4	45	1.7	28	–	–	–
PLATMC_75	37	2.0	27	–	–	–
PLATMC_155	22	2.1	25	–	–	–

Overall, a significant decrease in molar mass and increase in dispersity (Ð) were observed under both conditions (Figure 3 and Table 1) over the time period. Three samples were collected during printing and analyzed (Table 1). The results show that PLATMC had a 59% reduction in M_n_ during the first 4 min (sample 1). Subsequently, the extent of degradation slow down with an M_n_ decrease of 9% between 4 and 75 min (samples 1 and 2). The polymer remained printable up to 155 min, with an 80% decrease in M_n._ We observed a sudden decrease in viscosity after 155 min, and after this time printing was not possible since polymer had too low viscosity for printing.

The results from Figure 3a, where temperature and pressure were held constant, showed almost the same initial degradation pattern. A 57% decrease in M_n_ was required until it was possible to start printing, but the copolymer started to lose viscosity faster compared to the non-continuous printing, showing an almost 87% reduction after 75 min compared to the initial condition in which this reduction was only 68% (Figure 3a). This shows that high pressure (8.5 bar) led to faster degradation compared to the low-pressure range (2–6 bar) (Figure 3a) because Bioplotter^®^ uses compressed air to extrude the polymer, and there is direct contact between the polymer and the air, hence, a higher-pressure results in more O_2_ contacts with the polymer, leading to thermo-oxidative degradation. This is in agreement with previous research, which has shown that lactide-based copolymers undergo thermo-oxidative degradation at high temperatures.^44,45^ Considering the two conditions together, it is evident that high molar mass PLATMC needs to be degraded at least 57%, and that an M_n_ of 45–50 kg/mol is suitable for printing using the Bioplotter^®^. In general, a M_n_ suitable for the start of printing using the Bioplotter^®^ varies depending upon the polymer composition and its viscoelastic behavior. For instance, our previous findings showed that M_n_ was found suitable at 117 kg/mol, 71 kg/mol, 75 kg/mol, and 59 kg/mol for PLLA, PCLA, PLGA, and PDLGA, respectively.^31^

To understand and follow up on thermo-oxidative degradation during printing over time, we performed TGA in O_2_ by simulating the printing conditions. The results are depicted in Figure 3b. The results show that PLATMC experiences a 4.5% mass loss during an isothermal TGA run at 190°C for 240 min, which suggests the formation of low-molar mass compounds during thermal degradation. As reported in the literature, degradation in the copolymer is caused by a combination of different mechanisms, such as random chain scission and transesterification reactions, which depend on the copolymer composition.^46,47^ Thus, we wanted to understand the possible degradation mechanism and performed ^13^C NMR (data not reported) and SEC. These tests revealed that no microstructure changes occurred for the printed sample during continuous and non-continuous printing; neither did we observe any changes in average L_L_ and L_T_ block length overall. However, the increase in dispersity observed over time suggests that chain scissions triggered by the high temperature took place, leading to a decrease in M_n_ for the PLATMC copolymer.

Molar mass determines the physical properties of polymers: crystallinity, for instance, has an enormous effect on the printed scaffold’s degradation profile. We analyzed the thermal properties of the samples printed under non-continuous and continuous conditions. The DSC results are presented in Figure 3c and Table 1 (non-continuous printing). The results show that there was no melting peak present and that crystallinity had decreased to zero from 12%. This suggested amorphous nature of the printed scaffolds. We observed a decreasing trend in T_g_ when samples were printed for a longer time (Table 1), attributable to the decrease in M_n_ and increase in Ð. However, the low T_g_ value is an advantage after implanting scaffolds in the body, as the polymer would be in the rubbery state and hence resulting in a more pliable and softer scaffold, which is an important consideration when designing scaffolds for adipose tissue engineering.

Our results indicate that there is a need to set optimal printing parameters and the residence time of the polymer in the printing cartridge in order to control degradation and obtain a usable scaffold. Hence, further, for in vitro cell studies scaffolds were printed using a pressure of 6 bar and speed within the range of 8–10 mm sec^-1^.

### Mechanical properties of scaffolds determined using finite element analysis

We carried out FEA simulations to better understand how the mechanical properties of the printed scaffolds are affected by the degradation during printing and the scaffold design. We explored the relationship between molar mass and the scaffold’s structural and mechanical properties. Different scaffold designs, B90 and B45, were considered herein for simulations. Two scenarios were considered: (i) Identical; material’s mechanical property was considered identical throughout the scaffolds by considering that M_n_ has no impact on it. (ii) Variable; mechanical property decreases linearly with M_n_ decrease throughout the scaffold.

**Figure 4.**
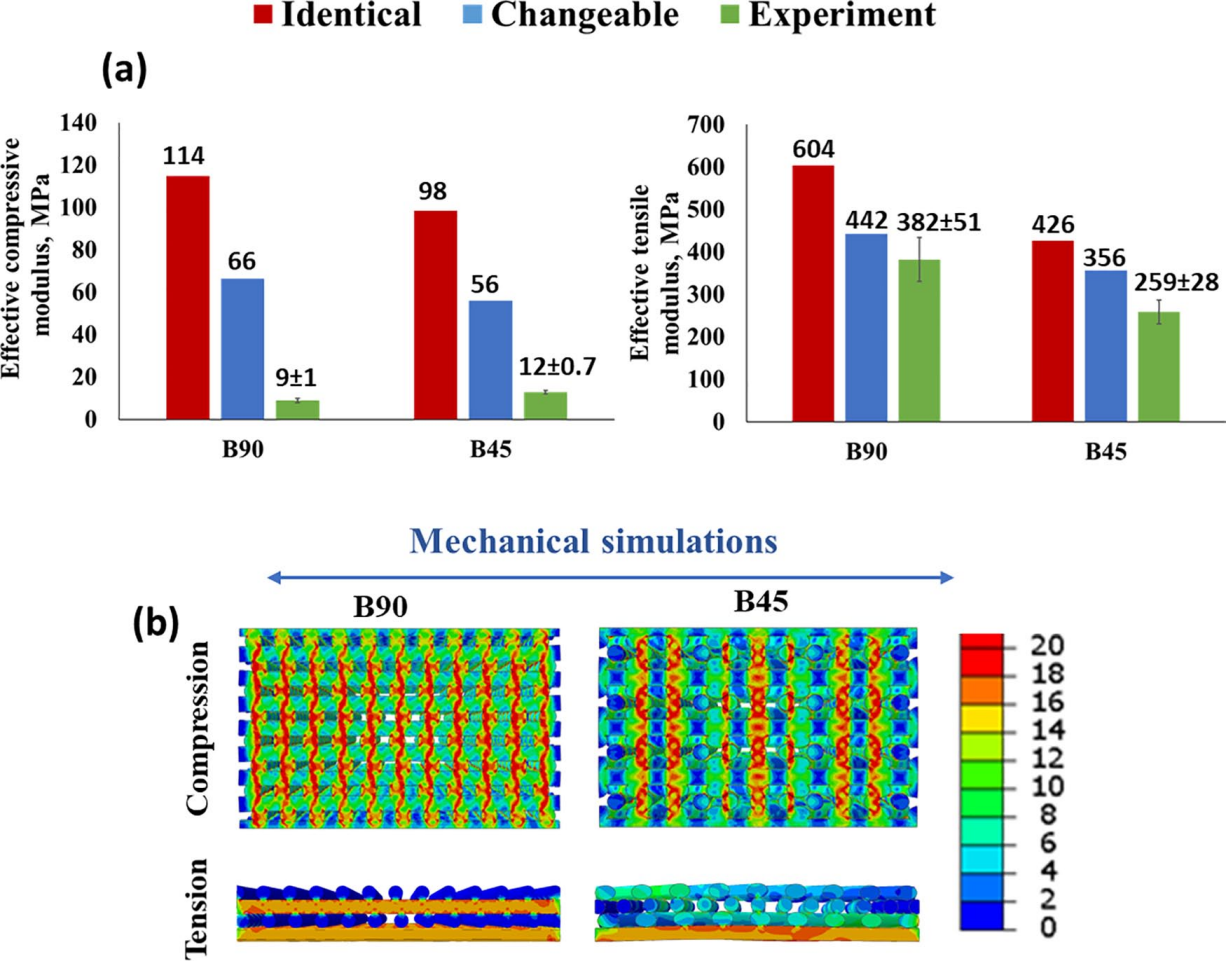
(a) The comparison between experimentally measured and FEA-computed compressive/tensile moduli for the B90 and B45 scaffolds. (b) FEA-based prediction of von Mises stress distribution in the strands of the scaffolds (middle cross-section view) under compression and tension in the identical case.

The results of the computational analysis (FEA) show that PLATMC degradation and scaffold design both have a clear impact on the mechanical properties of the scaffold, in both the compression and the tension stimulations. Figure 4a shows that the computed effective compressive/tensile modulus of the B90 scaffold is higher compared to that of the B45 scaffold, for both identical and variable cases. This finding is consistent with earlier studies.^40,48^ The reason for this difference is that the strand configurations determine the extent to which the scaffold design deforms in a given mechanical stimulation. As Figure 4b shows, the B45 distributed stress better and had fewer regions that exhibited high concentrations of stress throughout the deformed strands when compared to the B90 scaffold design. In addition, the variable case yielded a lower computed effective compressive/tensile modulus for both the B45 and the B90 scaffolds compared to the identical case. This observation is due to the fact that a softer material was assigned for the scaffold design in the changeable case. Consequently, the design’s mechanical resistance to deformation decreased and resulted in low scaffold stiffness.

Furthermore, we found the computed compressive/tensile moduli of the scaffolds show a better agreement with the measurements in the changeable case compared to the identical case. This observation indicates that the stiffness of the printed strands is no longer identical throughout the scaffolds instead decreases from the bottom section to the top section following the printing sequence. The reason can be attributed to the reduction in M_n_ of PLATMC during printing time. However, we noted a gap between FEA computed and experimentally measured compressive moduli for the compression case. The explanation to that is the stiffness assignment of strands in the compression model may not replicate the actual variation of the stiffness in printed scaffold samples. In the presented compression model, the scaffold is divided into 4 regions and a unique material stiffness is defined for each region and the magnitude of the material stiffness reduces from the bottom to the top region. Nevertheless, the stiffness distribution may be much more complicated than the description in the compression model considering the number of layers in the printed scaffold. Additionally, the strand stiffness may even vary in the same layer of the scaffold structure. Therefore, a further detailed study is needed to optimize the material stiffness assignment in FEA analysis to replicate the printed scaffold sample.

Despite the gap between the experimental and simulated values, it is obvious that it is important to optimize the printing process and choose the right printing window to obtain scaffolds without polymer degradation or at least to have control of it, and therefore, be able to control the mechanical properties of the scaffolds when using extrusion-based 3D printing. Meanwhile, it should be noted that this heterogeneous distribution of stiffness within the printed scaffold due to polymer degradation may influence the initial interactions between cells and strands and subsequent cells activity.

After establishing the printing parameters and subsequent characterization of polymer, we used the optimal printing conditions (low pressure range and early printing time) to fabricate the scaffolds for in vitro cell studies. The printed scaffolds had a resolution of 91% approximately, which was calculated using SEM images of the printed scaffolds and compared to the inner nozzle diameter. The scaffolds used in all the cell studies consisted of 4 layers unless otherwise stated, and were printed in the window between 8 to 25 min at 190°C. We then evaluated the printed scaffolds to validate the above-mention methodology and to see whether we had control over the target properties. We assessed both M_n_ and thermal properties, the printed scaffolds were amorphous, and the final M_n_ was 52.0 *±* 5 kg mol^-1^, such data can be correlated with results presented in Figure 3a and Table 1.

### Modification of 3D printed PLATMC scaffolds with PDA

After printing the scaffolds, we coated them with PDA to further improve their surface wettability.^36^ The PDA coating process occurs under basic conditions, pH of 8.5, in Tris-buffer. Because the PLATMC scaffolds might be affected at such an alkaline pH, we characterized the thermal properties and molecular weight of the PLATMC_PDA scaffolds. Our results showed no changes in the thermal properties of the scaffolds after modification, but we did find a significant reduction in M_n_ and an increase in Ð—27 ± 3 kg/mol and 3.5, respectively. Indeed, under a basic pH, hydrolysis can occur due to the formation of terminal hydroxyl ions and subsequently breaking of ester and carbonate linkages. This led to a decrease in M_n_ and the formation of low M_n_ compounds, as indicated by the increase in Ð from 1.8 to 3.5.^49,50^ However, the scaffold remained pliable and their geometry was unchanged (Figure S1).

#### Optimization of 3D printed PLATMC scaffolds

We conducted an in vitro cell study to choose the best design for coating with PDA. ASCs were seeded onto both the B90 and B45 scaffolds, and we then analyzed cell proliferation at day 7 and 11 and cell differentiation at day 21. The results showed that cells were attached and proliferated well on both scaffolds, but the B90 design had more DNA content on day 7 compared to B45 (Figure S2b). In fact, B45 had direct pores due to the design, whereas B90 had a strand shift or needle offset distance of 0.15 mm from layer three, which resulted in the adherence of a greater number of cells. Interestingly, on day 11 we did not observe any significant differences. We further evaluated adipogenic differentiation of the cells seeded on the B90 and B45 at day 21 in adipogenic medium using the AdipoRed^TM^ assay and no significant difference was observed between the B90 and B45 scaffolds. We hypothesized that B45 would show better ASC differentiation due to its lower tensile modulus and better stress distribution (Figure 4), which favors adipogenic differentiation, but this difference was perhaps not large enough to influence cell behavior under the evaluated circumstances. We then prioritized the seeding number, which was higher on B90, when selecting scaffold design to continue with.

#### Characterization of the modified 3D printed scaffolds

**Figure 5.**
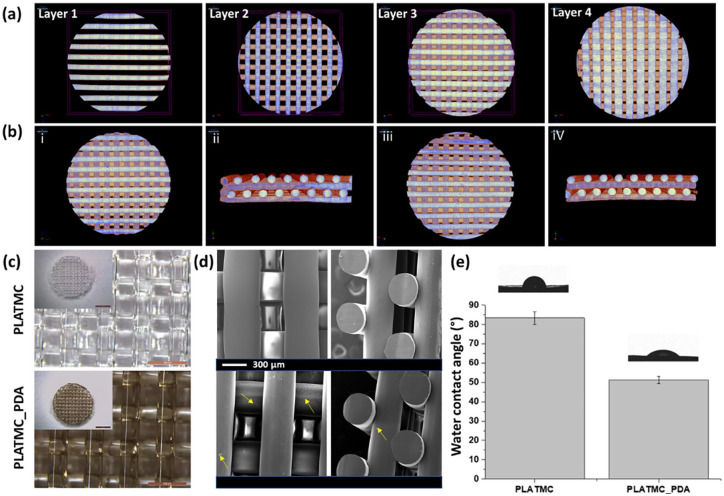
Characterization of the 3D printed scaffolds. Micro-CT images of 3D printed scaffolds: (a) 4 different printed layers; (b) printed scaffolds of PLATMC (i and ii) and PLATMC_PDA (iii and iv); (c) stereomicrograph of PLATMC and PLATMC_PDA, showing the visual difference before and after modification with PDA (scale bar- 500 µm), inset shows picture of whole scaffolds (scale bar: 2 mm); (d) SEM micrograph of printed PLATMC and PLATMC-PDA scaffolds. The PLATMC surface was smooth, whereas the PLATMC_PDA surface showed self-particulate (arrows) of DA. (e) Water contact angle measurements of the PLATMC and PLATMC_PDA scaffolds.

We used the B90 design with a slight modification of the scaffold’s geometry: the shifting distance was changed to 0.35 mm instead of 0.15 mm. In the initial design, the third layer was not exactly located in the gap between layers 1 and 2, but the 0.35 mm shift would place it completely in between layers 1 and 2 (Figure 5). This scaffold design strategy would increase adherence of the cells and result in higher seeding efficacy. The scaffold’s geometry and surface morphology were analyzed using Micro-CT and SEM (Figure 5). The results show that the scaffolds had well-connected strands and a porous architecture. The measured total porosity was 49 ± 0.4 and 46 ± 3% for the PLATMC and PLATMC_PDA scaffolds, respectively. No significant difference was observed after dopamine modification. Figure 5b and d show the cross-sectional view of the scaffolds, where the strand shift (0.35 mm) after layer 3 can be observed.

The PLATMC scaffold exhibited a smooth surface, but interestingly, we observed self-particulates of DA on the PLATMC_PDA scaffolds, which was in accordance with previous research.^51,52^

The degree of surface hydrophilicity can be directly assessed by measuring the water contact angle.^53^ We analyzed the effect of PDA treatment on the printed scaffolds and measured the water contact angle (Figure 5e). The result showed that the PLATMC films had a water contact angle of 83 ± 3° and displayed the hydrophobic nature of the copolymer. The PLATMC_PDA films showed a water contact angle of 51 ± 2°. This improved surface hydrophilicity was due to the presence of the PDA coating, which contains polar groups such as hydroxyl and amine that increase its interaction with water.^35^ Typically, improved surface hydrophilicity enhances protein adsorption, cell attachment and proliferation.^54,55^ Previous reports also suggested that PDA coating improved the surface wettability of various materials.^56,57^ However, PDA coating can be varied by changing the coating time, DA concentration, and reaction temperature, all of which can influence surface hydrophilicity.^36,58^

### Cell studies

#### Cell attachment, spreading, and proliferation

**Figure 6.**
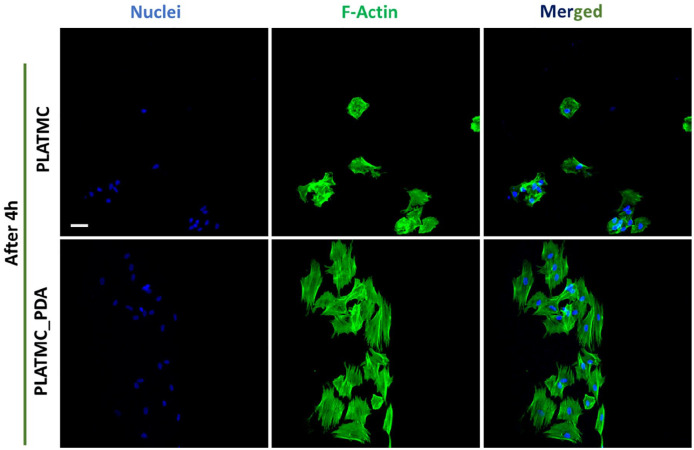
Confocal micrograph from the top of the scaffolds showing initial cell attachment and spreading on 3D printed scaffolds after 4h. Green = actin, and Blue = nucleus. (Scale bar- 50 µm). ImageJ software was used for background correction and better visualization of the cells.

In general, the biological process of cell adhesion to the material surface is a cascade that comprises several overlapping events, such as cell attachment, formation of cell-matrix adhesion or integrin binding, cell spreading, and actin cytoskeleton organization.^59,60^ The hydrophobicity or hydrophilicity of the biomaterial influences these cascade events toward cell adhesion. Previous research has shown that hydrophilic surfaces impart well-defined focal adhesion with the aid of protein adsorption, which subsequently affects cell attachment and proliferation.^36,61,62^ We assessed the initial cell attachment and spread after 4h of cell seeding by fluorescence staining of the F-actin and nucleus (Figure 6).

**Figure 7.**
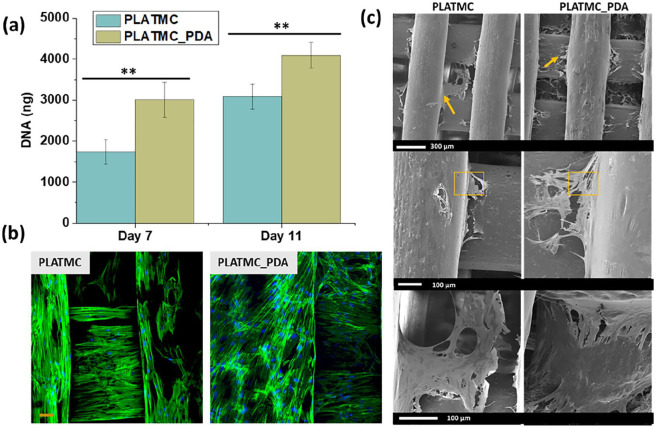
ASC response on the 3D printed scaffolds in growth medium. (a) DNA quantification using picogreen assay at days 7 and 11. (**p < 0.01) (b) Confocal images (day 7) showing cell distribution and proliferation on the scaffolds: actin (green), nucleus (blue), scale bar 50 µm. (c) SEM images were taken at day 7 (in growth medium) showing cell-material interaction on 3D printed scaffolds. Arrows show cell protrusion and the squares show the portion of the scaffold covered with cell sheet.

The results show that cells were poorly spread and had mostly a rounded shape on the PLATMC scaffolds, which is the normal behavior of cells on moderately hydrophobic surfaces, whereas on the PLATMC_PDA scaffolds the cells were well spread. Indeed, the PLATMC_PDA scaffolds had a comparatively more hydrophilic surface, which led to better cell attachment. Next, we assessed ASC proliferation onto the 3D printed scaffolds, evaluated cell proliferation by measuring DNA content at days 7 and 11. Figure 7a and b show the DNA quantification and confocal microscopy of the 3D printed scaffolds at days 7 and 11.

The result showed that cell numbers increased from day 7 to day 11 on both sets of scaffolds. However, a significantly higher cell number was observed on the PLATMC_PDA scaffolds compared to the PLATMC ones, both at day 7 and 11. The confocal micrograph provided visual proof and confirmed the presence of a higher number of cells on the PLATMC_PDA scaffolds. The enhanced proliferation on the PLATMC_PDA scaffolds is ascribed to the hydrophilic nature of the PLATMC_PDA scaffolds, due to the presence of polar bioactive functional groups (OH and NH_2_). It has been shown that a biomaterial surface with amine and carboxyl functional groups adsorbs more proteins compared to a methyl (CH_3_) surface.^63,64^ Furthermore, adsorbed proteins on the surface can affect cell-integrin binding and eventually regulate cell adhesion, proliferation, and differentiation.^65,66^ However, protein adsorption on biomaterial surfaces is highly influenced by many factors, such as hydrophilicity or hydrophobicity, surface chemistry, ionic and electrostatic interactions, intermolecular forces, and the charge of the biomaterial surface, and proteins change their conformation and interaction with surfaces accordingly.^67,68^ In addition, cell adhesion receptor proteins present on the cell surface, such as integrins, help cells bind to the substrates and regulate the fate of the stem cell.^69^ Focal adhesion kinase (FAK) is a protein that regulates integrin-mediated cell adhesion and spreading,^70^ and it has previously been shown that a PDA-coated surface enhanced initial adhesion and spreading of human adipose-derived stem cells and resulting in a greater expression of focal adhesion kinase (FAK) in the early stage of cell attachment. ^71^ Studies also suggested that FAK signaling may be vitally required for the adipogenesis in mesenchymal stem cells.^72,73^ Similarly, β_1_ integrin receptor expression also found to be higher on polydopamine coated surfaces which could be another reason for well-defined focal adhesion in ASC.^74^

We further analyzed the cell-material interaction using SEM, corroborating the higher number of cells with SEM images taken at day 7 (Figure 7c). The SEM images visually confirmed the presence of a higher number of cells on the PLATMC_PDA scaffolds compared to the PLATMC scaffolds. The images show that almost the entire scaffold was covered with cells, where a cell sheet that seems thicker on PLATMC_PDA scaffolds. Additionally, we found more cells entrapped in between the PLATMC_PDA scaffold strands compared to the PLATMC scaffolds, which may be due to the higher hydrophilicity of these scaffolds.

#### Adipogenic differentiation and gene expression

Adipocyte differentiation is a sequential process and involves several sequential changes at the cellular level. Pre-adipocytes, in the presence of chemical cues, stop proliferation and start to differentiate and become mature adipocytes, expressing specific genes and accumulating triglycerides (lipid droplets).^75,76^ We, therefore, evaluated the potential of 3D printed PLATMC and PLATMC_PDA scaffolds toward adipose tissue engineering by evaluating the gene expression of selected adipogenic genes such as PPARG, CEBPA, LPL, ADIPOQ, and PLP1 using q-PCR.

**Figure 8.**
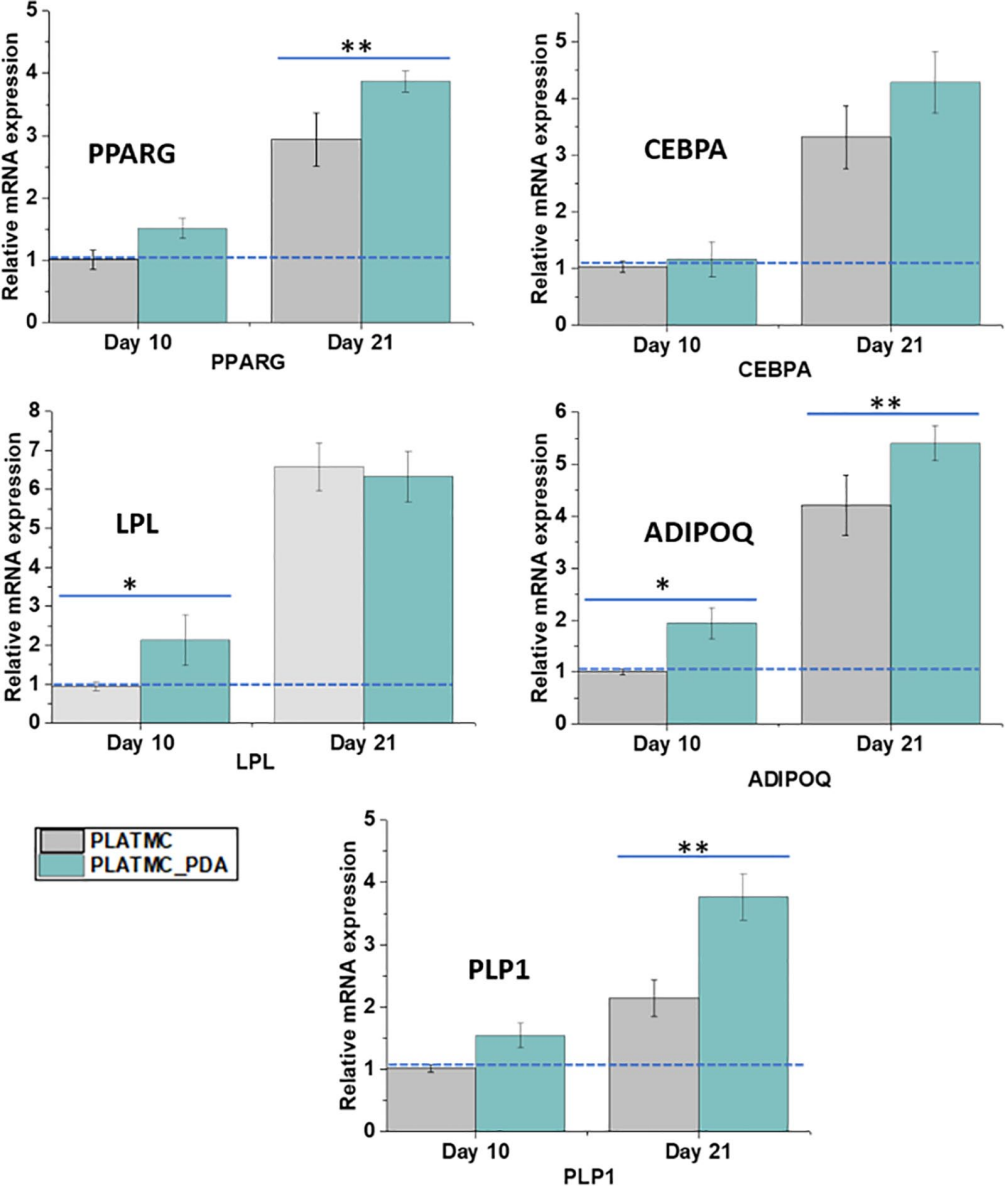
Relative mRNA expression of adipogenic genes at days 10 and 21 in adipogenic medium. Data are normalized to PLATMC scaffolds at day 10, compared to day 21, and GAPDH was used as an endogenous control for all the genes. *indicates a significant difference at the *p* < 0.05 level and **indicates a difference at the *p* < 0.01 level.

Figure 8 shows the relative expression of selected adipogenic genes from ASC cultured on the printed scaffolds, normalized to the PLATMC scaffold on day 10. A series of transcriptional factors are involved in adipogenic differentiation, in which PPARG and CEBPA play a key role but PPARG dominates over CEBPA.^77^

On day 10, both scaffolds showed expression of PPARG and CEBPA, with no significant difference between the two. These markers are expressed at the early stage of differentiation, and thus the presence of the expression of these genes means that these scaffolds support the adipogenic differentiation of the cells. However, expression of these genes increases throughout the process of differentiation, which initiates a positive feedback loop that induces its own expression and induces the expression of other adipogenic genes involved in the adipogenic differentiation process.^75,78^ In addition, PPARG induces expression of CEBPA and makes a regulatory loop by binding on the promoter region. Its cooperative function controls the other genes’ phenotypes, which induces the formation of the mature adipocyte phenotype from preadipocytes.^75,79,80^ Interestingly, on day 21 we observed a significant upregulation of PPARG in the PLATMC_PDA scaffolds compared to the PLATMC ones, which showed that the PLATMC_PDA scaffolds promoted adipogenic differentiation at this later stage.

We also evaluated the expression of LPL at day 10, this gene is secreted by mature adipocytes, and its mRNA expression at the initial stage has generally been considered an early sign of differentiation.^81^ It is essential in mature adipocytes for the deposition of intracellular lipids. We observed a higher expression of LPL in the PLATMC_PDA scaffolds at day 10, which corroborates the early differentiation of the cells present on PLATMC_PDA scaffolds. However, on day 21 there was no significant difference between the modified and unmodified scaffolds. This can be correlated with the expression of ADIPOQ, which was higher on days 10 and 21 in the PLATMC_PDA scaffolds compared to the PLATMC ones. The ADIPOQ gene encodes for adipokine, which secretes a protein hormone from adipose tissue that enhances adipocyte differentiation and lipid droplet formation.^81,82^

We also assessed the expression of perilipins (PLPs1-5), which are the proteins that are mainly associated with lipid droplets (neutral lipids). On day 10 we did not observe any significant difference between the PLATMC and PLATMC_PDA scaffolds, but interestingly, we found a higher expression of PLP1 in the PLATMC_PDA scaffolds compared to the PLATMC ones on day 21. Perilipins, especially PLP1, are abundantly expressed in mature adipocytes and can be considered a late marker of adipogenesis. PLP1 is present on lipid surfaces and is known to regulate hydrolysis and the accumulation of the triglycerides.^83,84^

**Figure 9.**
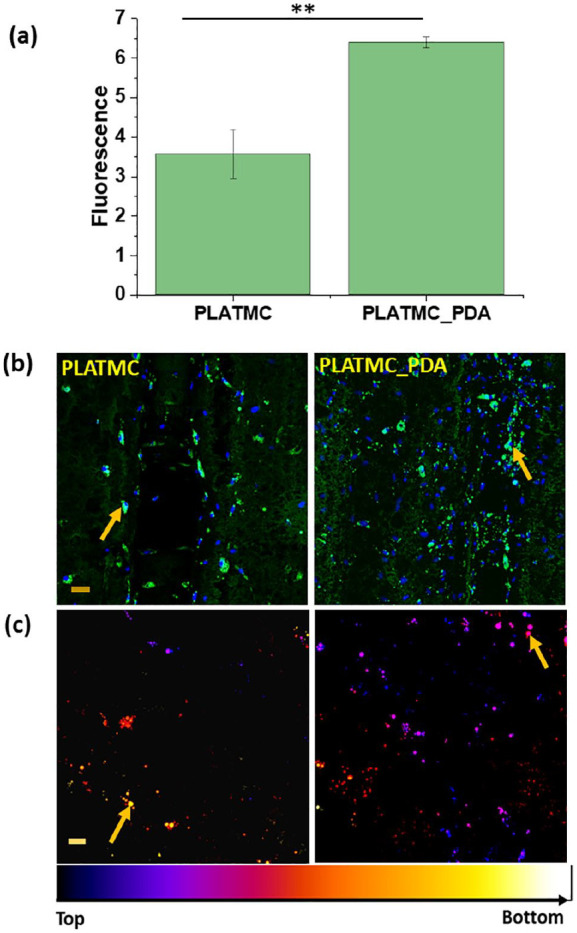
Adipogenic differentiation after 21 days of culture in adipogenic medium seeded onto the 3D printed scaffolds. (a) Quantification of lipid droplets (intracellular triglyceride) using AdiopoRed^TM^ assay (**p < 0.01). (b) Confocal images of the stained lipid droplets (green) and nucleus (blue). (Scale bar 50 µm (10x)) (c) Temporal color-coded representation of lipid droplets showing the distribution of lipid droplets on and inside the scaffolds (Scale bar 20 µm (20x)). Different colors were given using ImageJ.

To assess the late stage of ASC differentiation, we evaluated the accumulation of intracellular triglycerides to see whether PDA coating augments adipogenesis. ASC, in the presence of an adipogenic supplement or chemical cues, becomes mature adipocytes and deposit lipid droplets, which can be considered a marker of adipogenic differentiation.^75^ We quantified triglycerides accumulation using the AdipoRed^TM^ assay, after culturing cells in an adipogenic medium for 21 days (Figure 9). AdipoRed^TM^ is a solution of Nile red, which partitions in the presence of a hydrophobic environment and gives a fluorescent signal that can then be measured.^85^

Figure 9a shows the quantification of intracellular lipid droplets. The results demonstrate that cells cultured on the PLATMC_PDA scaffolds had a higher accumulation of lipid droplets compared to the PLATMC ones, a finding that was further confirmed by the confocal microscopy. The droplets were dispersed all over both types of scaffolds and could also be seen on the inside layer of the printed scaffolds. Over the time period, during terminal differentiation, small droplets fused together to form bigger droplets (indicated by arrows), which were seen more on the PDA-coated scaffolds (Figure 9b and c). This is corroborated by the higher expression of the PPARG, ADIPOQ, and PLP-1 genes at day 21 in adipogenic medium.

Taken together, these results suggest that both scaffolds support adipogenesis, but that the process was enhanced on the PLATMC_PDA scaffolds due to the PDA coating. Thus, this type of 3D device from medical grade polymer could potentially be used in adipose tissue engineering. However, in vivo exploration is warranted to develop it further.

## Conclusions

We developed soft, pliable, degradable 3D scaffolds using medical grade poly(L-lactide-co-trimethylene carbonate) in an effort to address the problem of adipose tissue regeneration in large-volume defects. 3D scaffolds were developed using a direct extrusion-based 3D printing method and the printability was established. The molar mass of the polymer was affected by the printing process, decreasing over the printing time period, and the scaffolds became amorphous. The results of computational simulations reveal the importance of limiting degradation during printing: gradual degradation resulted in scaffolds with mechanical properties varied across the printing time.

Scaffolds were then printed using optimal printing parameters (low pressure) in two design (B45 and B90), selecting a printing time window where degradation was minimal. Furthermore, in vitro cell-material interactions were assessed with both designs and the scaffolds with one design (B90) were subsequently modified with polydopamine. In the in vitro study, the seeded human adipose-tissue–derived stem cells attached, proliferated, and showed differentiation on both sets of scaffolds but this process was enhanced in the PLATMC_PDA scaffolds. Thus, the in vitro results suggest that PLATMC_PDA based scaffolds have the potential to serve as an implantable resorbable biomedical device for adipose tissue regeneration.

## Supplemental Material

Supplementry_information – Supplemental material for Engineering 3D degradable, pliable scaffolds toward adipose tissue regeneration; optimized printability, simulations and surface modification Click here for additional data file. Supplemental material, Supplementry_information for Engineering 3D degradable, pliable scaffolds toward adipose tissue regeneration; optimized printability, simulations and surface modification by Shubham Jain, Mohammed Ahmad Yassin, Tiziana Fuoco, Hailong Liu, Samih Mohamed-Ahmed, Kamal Mustafa and Anna Finne-Wistrand in Journal of Tissue Engineering
