# Supplementary material for: Engineering 3D degradable, pliable scaffolds toward adipose tissue regeneration; optimized printability, simulations and surface modification
Source: J Tissue Eng. 2020 Sep 16;11:2041731420954316. doi: 10.1177/2041731420954316 (PMC7498972; doi:10.1177/2041731420954316)
Supplement: Supplementry_information – Supplemental material for Engineering 3D degradable, pliable scaffolds toward adipose tissue regeneration; optimized printability, simulations and surface modification [file Supplementry_information.docx]

**Supplementary information for**

**Engineering 3D degradable, pliable Scaffolds Toward Adipose Tissue Regeneration; Optimized Printability, Simulations and Surface modification**


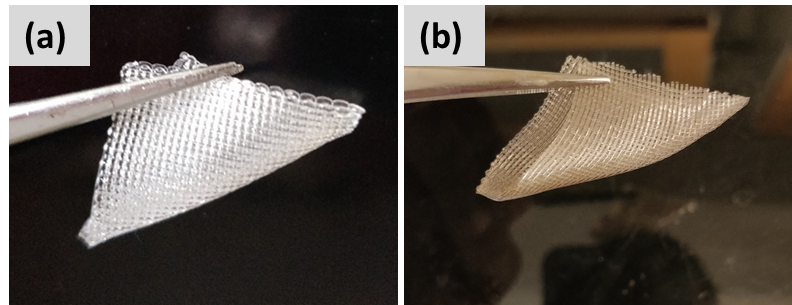


**Figure S1:** Digital picture of 3D printed sheet showing pliability (a) PLATMC (b) PLATMC_PDA


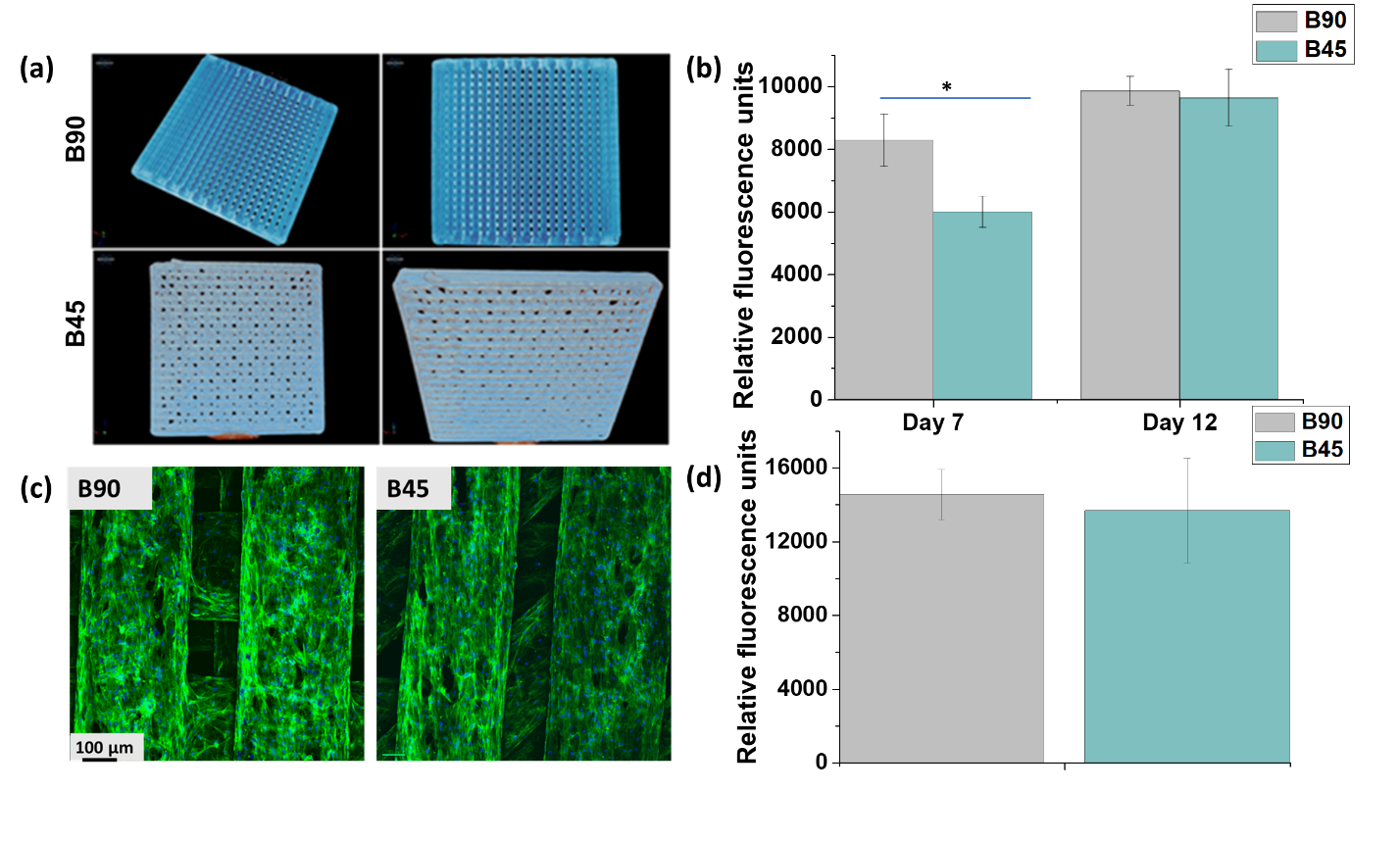


**Figure S2:** (a) Micro-CT images of printed scaffolds (b) DNA quantification of the ASC cultured on scaffolds at days 7 and 11. (c) Confocal micrograph of the ASC at day 11. (d) Quantification of the Intracellular triglyceride accumulation using AdipoRed^TM^ assay after 21 days culturing ASC in AM.

**Table S1:** Primers sequence used in the study.

| Gene | Gene ID | Amplicon length |
| --- | --- | --- |
| **GAPDH** | Hs02758991_g1 | 93 |
| **PPARG** | Hs00234592_m1 | 77 |
| **CEBPA** | Hs00269972_s1 | 77 |
| **LPL** | Hs00173425_m1 | 103 |
| **ADIPOQ** | Hs00605917_m1 | 71 |
| **PLP 1** | Hs00160173 | 54 |

**Table S2.** Characterization of the copolymer granules.

| **IV**  **(dL g−1) ^a^** | **M_n_ (kDa) ^b^** | **Ð ^b^** | **L-Lactide (mol%) ^c^** | **L_LL_ ^d^** | **L_T_^d^** | **T_g_ (°C) ^e^** | **T_m_ (°C) ^e^** | **X_c_ (%) ^e^** | **T_5%_ (°C) ^f^** |
| --- | --- | --- | --- | --- | --- | --- | --- | --- | --- |
| **1.4** | 109 | 1.6 | 60 | 2.3 | 1.5 | 29 | 158 | 12 | 280 |

^a^ Inherent viscosity (0.1% CHCl_3_) as provided by Evonik.

^b^ Number average molecular weight and dispersity obtained by SEC in CHCl_3_ using narrow polystyrene molecular weight distribution calibration curve.

^c^ Determined from ^1^H NMR in CDCl_3_.

^d^ L_LL_ and L_T_ represent the average block length of the L-lactide and trimethylene carbonate units, respectively, calculated from ^13^C NMR ^80^.

^e^ Data reported from the first run of DSC (T_g_, T_m,_ and X_c_).

^f^ Temperature at which 5% of mass loss was observed in a dynamic TGA run in O_2_ flow.
